# Septic Tibial Nonunions on Proximal and Distal Metaphysis—A Systematic Narrative Review

**DOI:** 10.3390/biomedicines11061665

**Published:** 2023-06-08

**Authors:** Konstantinos Sidiropoulos, Andreas Panagopoulos, Konstantinos Tsikopoulos, Alkis Saridis, Stelios F. Assimakopoulos, Antonis Kouzelis, Ioannis N. Vrachnis, Panagiotis Givissis

**Affiliations:** 1 Medical School of Patras, University of Patras, 26504 Patras, Greece; 2 Patras University Hospital, Orthopaedic Department, 26504 Patras, Greece; 3 404 Army General Hospital, 41222 Larissa, Greece; 4 General Hospital of Drama, Orthopaedic Department, 66100 Drama, Greece; 5 School of Health Sciences, Faculty of Medicine Department of Internal Medicine-Division of Infectious Diseases, University of Patras, 26504 Patras, Greece; 6 Aristotle University of Thessaloniki, 54124 Thessaloniki, Greece

**Keywords:** septic nonunion, infected metaphyseal tibia fractures, surgical options, efficiency

## Abstract

Background: Infected nonunion of the tibia represents a challenging complication for orthopedic surgeons and poses a major financial burden to healthcare systems. The situation is even more compounded when the nonunion involves the metaphyseal region of long bones, a rare yet demanding complication due to the poor healing potential of infected cancellous bone; this is in addition to the increased likelihood of contamination of adjacent joints. The purpose of this study was to determine the extent and level of evidence in relation to (1) available treatment options for the management of septic tibial metaphyseal nonunions; (2) success rates and bone healing following treatment application; and (3) functional results after intervention. Methods: We searched the MEDLINE, Embase, and CENTRAL databases for prospective and retrospective studies through to 25 January 2021. Human-only studies exploring the efficacy of various treatment options and their results in the setting of septic, quiescent, and metaphyseal (distal or proximal) tibia nonunions in the adult population were included. For infection diagnosis, we accepted definitions provided by the authors of source studies. Of note, clinical heterogeneity rendered data pooling inappropriate. Results: In terms of the species implicated in septic tibial nonunions, staphylococcus aureus was found to be the most commonly isolated microorganism. Many authors implemented the Ilizarov external fixation device with a mean duration of treatment greater than one year. Exceptional or good bone and functional results were recorded in over 80% of patients, although the literature is scarce and possible losses of the follow-up were not recorded. Conclusion: A demanding orthopedic condition that is scarcely studied is infected metaphyseal tibial nonunion. External fixation seems promising, but further research is needed. Systematic Review Registration: PROSPERO No. CRD42020205781.

## 1. Introduction

Managing an infected nonunion is an exacting task for orthopedic surgeons, not only as it poses a significant burden on patients and their families, but also due to the significant cost of healthcare systems [1,2]. In a recent systematic review of 17,073 patients with open tibia fractures, complication rates of infection, nonunion, and subsequent amputation were found to be 22%, 11%, and 16%, respectively [3]. On top of that, hospitalization cost varied from USD 428 to USD 151,832, with an average hospital stay of 56 days. Likewise, Hendrick et al. conducted a systematic review of 8110 participants with intramedullary (IM) nailing for a tibial shaft fracture, and reported nonunion and deep infection rates of 11% and 3%, respectively [4].

In a recent systematic review of 41,429 patients with tibial fractures, Tian et al. [5] defined the main predisposing factors of nonunion as follows: age >60 years, male gender, body mass index (BMI) >40, smoking, diabetes, nonsteroidal inflammatory drug (NSAIDs) or opioid user, fracture of the middle and distal tibia, high-energy fracture, open fracture, Gustilo–Anderson grade IIIB and IIIC, Müller AO type C, open reduction, fixation model, and infection. The authors also reported the prevalence of nonunion at 6.8% with closed reduction and minimally invasive percutaneous plate osteosynthesis as having the lowest risk of nonunion. Regarding infection rates, a machine learning algorithm to identify patients with tibial shaft fractures at risk of infection after operative treatment was published recently, identifying seven stratified risks: (1) Gustilo–Anderson or Tscherne classification, (2) bone loss, (3) mechanism of injury, (4) polytrauma, (5) AO/OTA fracture classification, (6) age, and (7) fracture location [6]. Metsemakers et al. [7] identified risk factors for deep infection and nonunion/malunion after intramedullary nailing in the tibia, but failed to identify a multifactorial model; polytrauma and primary external fixation were the only risk factors leading to nonunion and deep infection, respectively.

There is a paucity of literature on the incidence of infection and associated risk factors, especially for fractured distal and proximal tibia metaphysis. Parkkinen et al. [8] reported a 5.2% incidence of deep infection (82% acute) for 655 proximal tibial fractures treated with open reduction and plate fixation; 50% required muscle flap coverage, and 5 patients (15%) eventually underwent above-the-knee amputation. The main risk factors included age ≥50, obesity, alcohol abuse, OTA/AO-type-C fracture, and previous fasciotomy. Bleeker et al. [9] performed a systematic review of how we personalize surgery for the treatment of distal tibial fractures using intramedullary nailing or plate fixation; a total of 1332 patients were analyzed, including 10 randomized clinical trials (RCTs) (n = 873) and 5 observational studies (n = 459). Plating may lead to lower risk of malunion, but was higher for infection (8%). No differences were seen in nonunion, subsequent reintervention, and functional outcomes.

An infected tibial nonunion entails treatment for a significant amount of time and patient discomfort; establishing a correct diagnosis is of utmost importance. To elaborate, an internationally accepted definition of fracture-related infection (FRI) was recently adapted [10,11], with two levels of certainty around diagnostic criteria, i.e., confirmatory (infection is present) and suggestive (further investigation is required to exclude possibility of an FRI). Except for appropriate antibiotic treatment, key aspects of surgical management comprise thorough debridement, irrigation, fracture stability (usually with Ilizarov frames), dead space management, and adequate soft tissue coverage with or without bone grafting. Yet, outcomes are compromised by poor soft tissue status, actively draining sinuses, osteomyelitis, osteopenia, limb length discrepancy, stiffness, and contracture of adjacent joints [12,13,14,15,16,17].

Management becomes more complicated when the infected nonunion is in the proximal or distal metaphysis. The porosity of the cancellous bone differs from that of cortical bone due to differences in cellularity, rich blood flow, and increased contact area [18,19]. As such, the occurrence of metaphysical nonunion is more uncommon, but also more troublesome to treat, often accompanied by poor bone stock (osteoporosis), small metaphyseal bone segments, deformity, bone deficit, soft tissue lesions, and post-traumatic arthritis [18,19,20]. Functional results have not been studied sufficiently so far; therefore, the purpose of this study was to systematically review and map the current literature on articles addressing infected tibial nonunions at the proximal or distal metaphysis. Incidence, previous treatment, treatment protocol, antibiotic regimen, clinical and radiological outcomes, and complications highlighted our investigation.

## 2. Materials and Methods

The current systematic review was registered prospectively with PROSPERO (CRD42020205781). This review adhered to published principles and was recommended for meta-analyses of human studies, followed by PRISMA guidelines [21].

### 2.1. Inclusion and Exclusion Criteria

This systematic review considered experimental and quasi-experimental study designs, including randomized controlled trials, nonrandomized controlled trials, before and after studies (pre–post studies), and interrupted time-series studies. In addition, analytical observational studies (prospective and retrospective cohort studies, case-control studies, and analytical cross-sectional studies) were considered. Descriptive observational study designs, including case series, individual case reports, and descriptive cross-sectional studies, were also considered. Systematic reviews that met inclusion criteria were not excluded, depending on research questions. We included in vivo studies to explore treatment options and results of different techniques in cases of septic, active, or quiescent tibia nonunions (but not healed or treated metaphyseal (distal or proximal) tibia nonunions in adult populations, as epiphyses were closed). For infection diagnosis, we accepted definitions provided by authors of source studies.

Anatomic location of nonunions other than metaphysis, absence of infection, open epiphysis, and simultaneous involvement of the neighboring joint were exclusion criteria. Moreover, case studies and series with less than 5 patients were excluded to increase validity and credibility in reporting. Of note, we included only papers with extractable, quantifiable information. Cadaveric and animal studies were discarded.

### 2.2. Literature Search and Data Extraction

**Figure 1:**
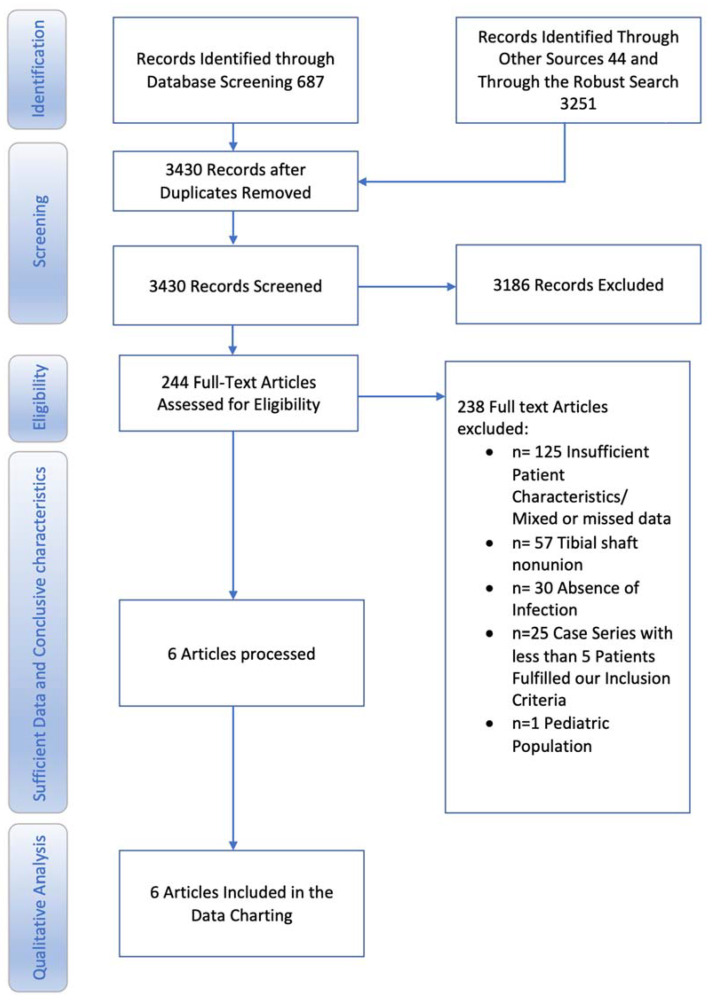
Flow chart of the systematic literature search according to PRISMA guidelines.

MEDLINE, EMBASE, and Cochrane Central Register of Controlled Trials (CENTRAL) was searched for completed studies published before 25 January 2021. We also considered the trial registries to search for completed yet unpublished studies. The search strategy was “(trial or random* or comparative study or case) and (septic or infected or osteomyelitis) and (nonunion or pseudarthrosis or late union) and tibia.” Moreover, a new robust search was conducted in October 2021 with search terms “infect* tibia nonunion” (Appendix A). KS and KT conducted the literature search independently without language restrictions. Records were deduplicated and remaining articles were examined with title and abstract screening, after which a full-text assessment was performed. Any discrepancies between authors in the study selection procedure were resolved through discussion. After removal of duplicates, the remaining 3430 articles were screened for eligibility. Following the abstract and title evaluation, 244 articles were found to be eligible for inclusion. In case of insufficient data, we attempted to contact authors at least twice, but with no results. Absence of infection and location of nonunion led to exclusion of 88 studies. Full texts were assessed, and 6 articles (published between 1995 and 2021) were ultimately included for qualitive synthesis. (Study Flowchart, Figure 1).

KS and KT independently extracted relevant information from included full-text articles, including score assessments pre- and postoperatively. In cases in which infected and noninfected tibia nonunions were included, we assessed data based on treatment in the presence of pathogens. Data extracted involved details about participants, concepts, context, methods, and key findings to septic metaphyseal nonunion of the tibia (Supplementary File).

### 2.3. Quality Appraisal

Two reviewers (AP and KS) assessed the quality of the included studies with the Moga Score, risk of bias tool, for case series developed using a Delphi approach [22]. The following domains were assessed: a clear statement of the aim of the study, a description of participant characteristics, single- or multicenter studies, eligibility criteria for entry into the study, consecutive recruitment of participants, entrance into the study at a similar point of the disease, clarity of co- and normal intervention, and competing interests and sources of support. Regarding the outcome measurements, a clear definition was requested when the data were collected and measured by the objective methods. Finally, statistical analysis (test, reported length and losses of the follow-up, adverse effects, random variability in the data analysis, and conclusion, which was supported by the results) was assessed. Each entry was evaluated as having each of the previous criteria or not. We assessed risk of bias across studies with any of 14 or more positive responses (>70%) of acceptable quality (low and moderate risk bias).

### 2.4. Outcome Assessment

The primary outcome measure was efficacy of the treatment methods, as measured by bone healing, infection eradication, and functional scores. Secondary outcomes included assessment of complications and limb length discrepancies. Complications defined by authors included reoperations, treatment method changes, and amputations.

Heterogeneity across characteristics of source studies and variability in baseline patient data rendered data pooling inappropriate. As a result, a pure qualitative analysis was deemed to be optimal in this systematic review.

## 3. Results

Clinical heterogeneity rendered data synthesis meaningless, so a narrative description of results was performed only. Mean participant age (44.6 years) and superiority in number of males (33 vs. 18 females) refer to a population of 51. In this population, except for 6 participants in Meselhy et al.’s study [23], the frequency of septic proximal metaphyseal tibial nonunion was lower (3 out of 45 individuals). The oldest study was published in 1995, while the newest was published in 2021. This 26-year window reflects the clinical practice in such a manner to analyze the management of this rare condition over the course of the years.

In the included studies, 51 individuals with infected tibia nonunions in distal or proximal metaphysis were analyzed. Of note, most studies included information on the microbial etiology. However, information on antibiotic susceptibility of the isolated pathogen(s) was not provided, except for Staphylococcus aureus strains, which were further characterized as methicillin-susceptible (MSSA) or as methicillin-resistant (MRSA). Study characteristics are presented in Table 1 [20,23,24,25,26,27]. Regarding treatment options, four studies implemented the Ilizarov method, one used the induced membrane technique, and Yoon et al. applied a staged combination. These treatments are not representative, but the literature is limited.

An overview of results could include how the most affected tibia metaphysis is distal. Most participants were diagnosed with distal tibial osteomyelitis, given the fact that the commonly involved region was the distal metaphysis. MRSA, MSSA, and coagulase negative staphylococci such as epidermidis and hominis were found to be the most common pathogens (32 out of 48 individuals for whom there is a registry), followed by pseudomonas aeruginosa and Acinetobacter baumannii. According to the Application of Methods of Ilizarov (ASAMI) [28], post-treatment functional scores were excellent or good (35 out of 42 participants for whom there is a relevant registry).

Brinker and O’Connor (2007) [24] conducted a prospective study with patients over 60 who had tibial nonunions treated with the Ilizarov method. This cohort consisted of 23 patients, of whom only 7 were diagnosed with distal proximal metaphyseal osteomyelitis. The pathogen was staphylococcus aureus for six of them and Staphylococcus Hominis for one; no plastic flaps were used in any case. Yet, the use of autograft was deemed appropriate for all except one. In five cases, the Ilizarov method was used for gradual deformity correction and compression, and in the other two for bone transport. The average follow-up was 30 months (range 18 to 61 months), with the loss of a patient due to cardiovascular disease 3 months later. In a description of postoperative complications, two patients developed cellulitis treated with intravenous antibiotics, with one developing pin site infection. Self-reported quality of life, the AAOS Lower Limb Core Scale, and the Short-Form 12 Physical Component Summary improved at the end of treatment, while the average of the Brief Pain Inventory Interference and Intensity items decreased. The Time Trade-Off quality of life tool improved for the whole cohort.

Eralp et al., through a retrospective case series with 16 to 70 months follow-up, recruited 13 patients (9 males and 4 females) with infected distal metaphyseal tibial nonunion [20]. Radical debridement with a vancomycin-loaded spacer, followed by the Ilizarov method, was performed on 10 patients, while a combined method (ankle arthrodesis and the Ilizarov method) was applied to another 2 patients, and 1 received the Taylor Spatial Frame for ankle arthrodesis. Isolated pathogens were staphylococcus aureus (8/13), coagulase negative staphylococcus (CoNS) (3/13), and pseudomonas aeruginosa with Escherichia coli (2/13). For bone grafting of the docking site, five patients received bone grafts and three received plastic flaps (two local fasciocutaneous flaps and one free latissimus dorsi flap). According to Paley, bone healing criteria were excellent and good in 11/13 patients, while the same number also had excellent or good functional results. It should be noted that union was achieved in all but one patient, with the mean postoperative difference in the American Orthopedic Foot and Ankle Society (AOFAS) score [29] ranging from 40 to 92k with a mean of 68.8. Among the reported complications, the usual tended to be pin track infections (n = 7), ankle stiffness (n = 3), and knee stiffness (n = 1), along with equine deformities (n = 3).

Lonner et al. retrospectively studied 10 patients with distal metaphyseal nonunion. Six cases were infected with unknown pathogens [25]. The follow-up was 48 months (26 to 81), and no bone grafts or plastic flaps were used. The Ilizarov method was applied after resection of the nonunion, with compression in three patients (two males and one female), compression only without excision in one male, and ankle fusion after excision in two patients (one male and one female). Five nonunions were treated without deformity in four, with excellent or good functional scores. The nonhealed nonunion showed reduced leg length discrepancy.

In a retrospective study published by Meselhy et al. (2021), six patients (four male and two female) with infected nonunions were presented. The inclusion criterion was shortening of less than 5 cm [23]. Treatment strategy was antibiotic therapy according to preoperative cultures without any debridement; the Ilizarov device was applied with two consecutive cycles of distraction and compression (accordion maneuver). No bone graft was used and pathogen duration of antibiotic treatment was unknown. With a follow-up of 28.5 months on average, fracture healing was achieved, and the functional result, according to Johner and Wurth, was excellent in four patients and good in the other two.

Siboni et al., through a retrospective study (10 patients: 6 males and 4 females) with metaphyseal nonunions (9 distal and 1 proximal) concluded that the induced membrane technique after thorough debridement and appropriate antibiotic therapy was effective for 6 of them [26]. The definitive treatment was a locking plate (five patients) and plaster cast (one patient) with a great deal of bone graft. Regarding failures with this technique, two patients achieved bone healing after a revision of internal fixation, removing the intramedullary nail with a locking plate. The other two were prompted towards ankle fusion and the other towards amputation. The mean follow-up was 34 months, while the mean Western Ontario and McMaster Universities Osteoarthritis Index (WOMAC) score, according to the authors for the whole cohort, was 23 +/−22.

Yoon et al. (2021) conducted a prospective study (nine males and one female) with tibia metaphyseal-infected nonunion individuals presenting a three-stage treatment strategy [27]. The first stage comprised the removal of all internal fixation and radical fixation. The bone gap was filled with gentamicin bone cement and stabilized with a monoaxial external fixator. The induced membrane technique was used with massive bone grafting (second stage). Infection eradication was confirmed when the intraoperative polymorphonuclear count was <10/high-power field. The definite treatment was distraction osteogenesis with the Ilizarov method or bone transport over a nail or plate when the soft tissue envelope became healthy. Bone graft was always used with a known pathogen. According to the Association for the Study and Application of Methods of Ilizarov (ASAMI), a classification [28] score bone result was excellent in 6, good in 3, and fair in 1. The functional ASAMI score was excellent in 4, good in 5, and fair in 1, with a follow-up performed over one year.

### Quality Assessment

Most domains were considered to have a low risk of bias, apart from the entrance time point, which was found to vary for each participant; more than one enrolled center, as well as consecutive recruitment, was absent in four out of the six included studies. We might mention that all six studies appear to be at a low risk of bias according to the Moga score for case series (>14 positive answers for the 18-point checklist).

## 4. Discussion

Infected tibia nonunion has a solid definition: a post-traumatic bony wound with an ununited fracture, compromised soft tissue envelope, and blood supply [30]. The clinical picture for a given patient suffering from infected tibial nonunion features pain, paradoxical motion of the fracture site, edema, and elevated inflammation markers. These are the common characteristics of the condition, while all other patient-related aspects are variable, as reflected by the results of the current study. For instance, the number of surgeries, extension of the debridement, type and duration of local or systemic antibiotic therapy, and bony reconstruction stabilization differ a lot among individuals. Each nonunion case has unique features due to a wide variety of patient characteristics, fracture patterns, and germ behaviors. There are no unambiguous guidelines or protocols regarding treatment options, duration, timelines, and antibiotic therapy. Patient data are lacking most of the time; no patient-reported outcomes are available, and the need for a large-scale multicenter RCT is eminent to detect indications and a possible guideline algorithm. Provided that the induced membrane technique or Ilizarov principles (including the correct size of frames, accurate tension and orientation of wires, and the use of olive wires for bending stiffness [31]) have been followed meticulously, an external fixation technique or combined method may be a reliable option for addressing tibia stabilization and fracture union with bone transport techniques [32].

In this review, we noticed that the major limitation was the scarce literature and the significant heterogeneity of the included studies. The limitation, based on the exclusion of case series with fewer than five participants, was of importance for us, as a low volume of surgeons and centers could affect the credibility of this report. This cut-off point is arbitrary in order to maintain balance between the risk of bias and the validity of results. The number of patients identified in the literature, related to septic tibial metaphysis, was 51 in total, which shows great diversity in the type and methods of treatment in the follow-up period, and without always having a control group to make necessary comparisons and produce correctly statistically significant results. This fact affects the level of potential bias and the possibility of safe conclusions to be conducted. As mentioned before, septic tibial metaphyseal nonunion as a clinical entity is rare. This could lead to a high risk of bias. On the other hand, patients with this condition need special treatment methods to achieve at least good functional and bone results. Moreover, a lack of data regarding pre- and postoperative limb length discrepancy and functional and quality-of-life scores do not facilitate any critical discussion about the usefulness of these methods for everyday life and the functional ability of patients.

Regarding measurements (scores) preoperatively and postoperatively to judge the clinical and functional outcome is absent from some studies, such that their distribution is not uniform. There was, as expected, a wide variety of germs that promote infections to be eradicated; apart from extensive or limited surgical cleaning (debridement), no treatment information was provided. Through the included studies (4 retrospective and 2 prospective, with only 17 participants), the use of autologous or nonautologous grafts for creating flaps vary in method and indication, without clear details about the application. A frequently reported conclusion is that treatment of septic tibial metaphysis is demanding, but there are not enough patients and studies to draw safe conclusions about the methods and treatment stages. A possible alternative is amputation of the limb, although there is a need for a carefully designed prospective study to treat septic pseudoarthrosis of the tibia, with extensive surgical cleaning of the soft and bony structures, placement of the Ilizarov device, and application of distraction osteogenesis.

There is ambiguity in the literature regarding risk factors that cause nonunions after long bone fractures. Thus, type of fracture, fracture site (tibia has an elevated risk for nonunions), age, smoking, and diabetes [33] appear to be implicated in this condition. An established infection results in nonunion regardless of other risk factors [34]. In severe injuries of tibial metaphysis, patients’ activity levels are compromised without significant differences in functional and clinical outcomes of limb salvage and early amputation at two years post-injury, presenting objective and functional outcomes below normal levels [35]. Viable treatment solutions for infected metaphyseal tibial nonunions could be debridement, antibiotics, and distractive osteogenesis with the Ilizarov method, given the basic principles of Paley for all problems (potential expected difficulties fully resolved nonoperatively), obstacles (potential expected difficulties fully resolved operatively), and complications (nonresolved and non-expected difficulties after the treatment period and during the early post-treatment stage) in the treatment period, such as muscle contractures, joint laxation, axial deviation, neurovascular injury, premature/delayed consolidation, pin site problems, refracture, and joint stiffness [36]. These could be local or systematic, intraoperative, early or late, and related to lengthening during distraction and during the fixation period. Excellent surgery with the right indications, a close follow-up of the patient, and nonpremature removal of the Ilizarov frame positively affect the result with few (or fewer) complications. Regarding tibial nonunions (in general, not only tibial metaphysis), segments of bone are created through corticotomies, distally or proximally, and bone healing is achieved through compression of the nonunion site after debridement of devitalized tissue [28]. The final outcome is a synthesis of two aspects: bone and functional results. Bone results are excellent when union is achieved without any recurrency of infection, deformity of less than 7 degrees (each axis), and tibia length discrepancy of less than 2.5 cm. Bone union with any two others of the above is good result; bone union with only one of the above characteristics is a fair result; and no bone healing is a poor result [28]. On the other hand, an excellent functional result is present when significant limp, ankle equinus rigidity, soft tissue dystrophy, pain, and inability to work are absent. When one of the above is present, the result is good, and so forth [28]. A recent retrospective analysis of complications of the bone transport technique using the Ilizarov method and distraction osteogenesis, including bone defect of femur (62 cases) and tibia (220 cases) due to fracture, osteomyelitis, and septic (15 tibias) and nonseptic nonunion, revealed that the most frequent complications were pin site infection (66%), axis malalignment (41%), joint stiffness (24%), soft tissue incarceration (22%), and delayed union on the docking site (13%) [37]. Other possible complications were delayed union or consolidation, nonunion, and refracture. Less soft tissue coverage of the tibia in lower-BMI patients (within the normal range), smaller defect size, and smaller external fixation time negatively affect the pin site infection prevalence. Axial deviation is more prominent in older patients and in the metaphyseal part of the long bones. So, patient age, defect size, and external fixation time are proportional to a high complication rate. Patients with lower socioeconomic status or mental illness are inappropriate candidates for distraction osteogenesis via the Ilizarov method. It is worth emphasizing that even in challenging situations, after tibial fracture or deformity, the Ilizarov method shows excellent results, even after 15-to-30-years’ follow-up [38]. Only 4 patients out of 72 required amputations, while 5 more needed ankle arthrodesis. Almost half of the complications (knee stiffness, infection, motor, or sensor nerve deficit) occurred during the first two years.

While distraction osteogenesis is based on bone basic science and the ability of periosteum regeneration under tensile forces, when the surrounding microenvironment is preserved (minimal invasive techniques, no infection, no compromised blood supply), the induced membrane technique is founded on the tissue response to any foreign body (foreign body tissue reaction) [39]. The newly formed membrane is highly active. Initially, extensive debridement and infection eradication are required. Secondly, the bone defect is filled with a cement spacer, and after a few weeks, bone graft replaces the removed spacer. Special characteristics of the spacer, bone fixation, time of the second stage, spacer removal, and bone graft affect bone healing and the result. An interval of six to eight weeks between the two stages, antibiotic-impregnated bone cement, and the use of internal fixation in the second stage lower the complication rate of this technique [40]. The most frequent complications are nonunion and infection persistence or appearance. To be more precise, regarding the present study, while the ultimate outcome remains the same (infection eradication, bone and soft tissue envelope healing), these two techniques use different approaches to achieve the same result, and there are few data available when tibial metaphysis is the location of septic nonunion.

Regarding the use of bone grafts, the best union rates (i.e., 90%) are achieved when placed within six to eight weeks postoperatively [41]. Bone graft use before infection eradication can compromise treatment. Instead of bone graft, materials with osteoconductive properties to prevent bacterial growth, such as bioactive glass (S53P4/BonAlive BioM), are effective in bone regeneration due to their ability to interact with the body to form new bone [14,42].

## 5. Conclusions

Septic metaphyseal tibial nonunion is a rare condition with limited treatment strategies available, compromising patients’ quality of life. Functional and objective results without treatment can be similar to limb amputation. No consensus on treatment protocols were reached, with the relevant literature being scarce and of moderate quality. This study shows that the application of an Ilizarov external fixation device and an induced membrane technique following debridement, coupled with systemic antibiotic administration, yields an improvement on infection-related outcomes as measured by eradication rates, fracture union, and functional improvement. Future authors should conduct prospective patient-reported outcome measures (PROM) studies with sufficient follow-up and sample sizes to bridge the literature gap.

## Figures and Tables

**Table 1:** Study characteristics.

Study (Year)	Number of Patients Treated	Method	Plastic Flaps	Bone Graft	Postoperative Score Assessment	Microbe	Follow-Up
Brinker (2007)	7 infected nonunions (3M 4F), all distal prospective case series	Ilizarov method 5/7 gradual deformity correction and compression, 2/7 bone transport	None	6/7 autograft	AAOS Lower Limb Core Scale, Brief Pain Inventory, Medical Outcomes Short-Form 12, Time Trade-off (QALY)	Staph Aureus 6/7 (MSSA), 1/7 Staph hominis homini	Average 38 months (18–61 months); 1 patient died 3 months after treatment
Eralp (2016)	13 patients (9M 4F), retrospective case series	Radical debriment with cement vancomycin-loaded spacer for 2 weeks, then Ilizarov or combined method (Ilizarov plus nail for ankle arthrodesis, 2 patients), 1 patient ankle arthrodesis with Taylor spatial frame	1 patient free latissimus dorsi flap, 2 local fasciocutaneous flaps	5 patients, docking-site grafting	Paley bone healing criteria: 5 excellent, 6 good, 2 fair. Paley functional criteria: 10 excellent, 2 good, 1 poor AOFAS scores	8 staph aureus (MSSA), 2 pseudomonas aeruginosa/*E. coli*, 3 CN staphylococcus	16–70 months
Lonner (1995)	6 infected nonunions (4M 2F)	Ilizarov frame with resection of the pseudarthrosis and compression in 3 patients (2M 1 F), compression without excision in 1 male patient, and ankle fusion after excision in 2 patients (1M and 1F)	None	None	5 healed (4 of them without any deformity). 1 had 5 degrees varus. Functional score (Mezut) and functional class excellent in 4, good in 1, and poor in 1 (not healed, with deformity and limb discrepancy 4 cm)	Unclear	48 months (average) 26–81
Meselhy (2021)	Retrospective study, 6 patients (4M and 2F) with infected nonunion (active) with <5 cm shortening.	No debridement, antibiotics according to perioperative culture. Ilizarov external fixator and 4 cycles of distraction and compression (accordion maneuver)	Previous surgeries, 4/6	None	Hammer classification for fracture healing (2 1st class and 4 2nd class) Outcome score/result according to Johner and Wurth: 4 excellent; 2 good	unknown	28.5 months on average
Siboni (2018)	Retrospective study 10 patients (6M and 4F), 1 proximal and 9 distal metaphyses	Induced membrane, two-stage	Unknown	10/10	6 unions, 4 nonunions. From them, 3 united after one extra procedure, 2 with plate, 1 ankle arthrodesis, and 1 amputation. Mean WOMAC score 23 +/−22	*S. epidermidis*, *S. lugdunensis*, *S. capitis*, *S. warneri*, Staph. Aureus (MSSA), *S. hominis*, Staph. Aureus (MRSA)	Mean follow-up: 34 months
Yoon (2021)	Prospective study with 10 patients (9M and 1F) with infected nonunion on 8 distal and 2 proximal metaphyses.	First stage: removal of all internal fixation and radical fixation. Second stage: Induced membrane technique with massive bone grafting. Infection eradication was confirmed when intraoperative polymorphonuclear count was <10/high-power field. Third stage: Dist. osteogenesis with Ilizarov method or bone transport over a nail or plate when the soft tissue envelope was healthy.	Unknown	10/10	ASAMI: 1 fair, 5 good, 4 excellent	2 Enterobacter cloacae, 4 Staph. Aureus (MRSA), 3 Acinetobacter baumannii, Staph aureus MSSA, Pseudomonas aeruginosa	Over one year

## Data Availability

Research data, included partially in the Supplementary File, are available to anyone. Please contact the corresponding author for any clarifications.

## Supplementary Materials

The following supporting information can be down-loaded at: https://www.mdpi.com/article/10.3390/biomedicines11061665/s1, Supplementary File: Data extracted.

## Appendix A

### Search Strategy

March 2020: ((trial [Title/Abstract] OR random*[Title/Abstract] OR comparative study[Title/Abstract] OR case[Title/Abstract]) AND (septic[Title/Abstract] OR infect*[Title/Abstract] OR osteomyelitis[Title/Abstract])) AND (non-union[Title/Abstract] OR pseudarthrosis[Title/Abstract] OR late union[Title/Abstract] AND tibia*[Title/Abstract]) for PubMed/MEDLINE

January 2021: ((trial [Title/Abstract] OR random*[Title/Abstract] OR comparative study [Title/Abstract] OR case[Title/Abstract]) AND (septic[Title/Abstract] OR infect*[Title/Abstract] OR osteomyelitis[Title/Abstract])) AND (non-union[Title/Abstract] OR nonunion[Title/Abstract] OR pseudarthrosis[Title/Abstract] OR late union[Title/Abstract] AND tibia*[Title/Abstract]) for PubMed/MEDLINE. Similar search strategy was used for EMBASE and CENTRAL Libraries.
