# Supplementary material for: Septic Tibial Nonunions on Proximal and Distal Metaphysis—A Systematic Narrative Review
Source: Biomedicines. 2023 Jun 8;11(6):1665. doi: 10.3390/biomedicines11061665 (PMC10296242; doi:10.3390/biomedicines11061665)
Supplement: Supplementary file 1 [file biomedicines-11-01665-s001.zip › biomedicines-2284492-supplementary.pdf]

Patient	Sex	Age	Time from to presentation (months)	Number of previous surgeries	Tibial Region	Microbe	Treatment Method (mode)	Bone Graft	Plastic Flap	Days to union	Months to Final Follow-up	Complications	Results	Preop LLD (cm shortening of the affected limb)	Postop LLD (cm shortening of the affected limb)
**Br.1**	M	61	3	1	Distal	Staph. Aureus	Gradual Deformity correction + Compression	Autograft	None	432	47	Cellulitis	Excellent	Unknown	Unknown
**Br.2**	F	65	3	2	Distal	Staph. Aureus	Compression	None	None	90 (Death)	Death	Death	Death	Unknown	Unknown
**Br.3**	F	65	11	5	Distal	Staph. Aureus	Bone Transport	Autograft	None	427	19	None	Fair	Unknown	Unknown
**Br.4**	F	67	37	6	Distal	Staph. Aureus	Gradual Deformity correction + Compression	Autograft	None	283	61	Pin site infection	Good	Unknown	Unknown
**Br.5**	M	70	10	1	Distal	Staph. Aureus	Gradual Deformity correction + Compression	Autograft	None	217	43	Cellulitis	Very good	Unknown	Unknown
**Br.6**	M	77	25	2	Distal	Staph. Aureus	Bone Transport + tibial/calcaneal fusion	Autograft	None	587	35	None	Good	Unknown	Unknown
**Br.7**	F	78	11	1	Distal	Staph. Hominis Hominis	Gradual Deformity correction + Compression	Autograft	None	239	31	None	Good	Unknown	Unknown
**Er.1**	M	43			Distal	Inconclusive	Gradual Deformity correction + Compression			270	20	Pin site Infection	Excellent	1	3
**Er.2**	M	46			Distal	Inconclusive	Bone Transport			150	39	Pin site Infection, Equinus Deformity	Good	2	0

**Er.3**	M	63			Distal	Inconclusive	Gradual Deformity correction + Compression, Arthrodesis			210	20	Pin site Infection, Ankle Stiffness	Good	1	2
**Er.4**	M	35			Distal	Inconclusive	Gradual Deformity correction + Compression			210	36	Pin site Infection, Equinus Deformity, Ankle Stiffness	Good	2	3
**Er.5**	M	71			Distal	Inconclusive	Ankle Arthrodesis (Combined Technique)			135	63	Pin site Infection, Equinus Deformity, Knee Stiffness	Good	0	0
**Er.6**	M	53			Distal	Inconclusive	Bone Transport			300	18	None	Excellent	4	0
**Er.7**	M	66			Distal	Inconclusive	Compression			120	16	None	Good	0	0
**Er.8**	F	79			Distal	Inconclusive	Bone Transport			255	36	None	Good	1	1
**Er.9**	M	41			Distal	Inconclusive	Bone Transport			200	24	Pin site infection, malalignment >5	Fair	2	2
**Er.10**	F	38			Distal	Inconclusive	Ankle arthrodesis (Taylor Spatial Frame)			270	51	Revision ankle retrograde IMN	Fair	2	4
**Er.11**	F	28			Distal	Inconclusive	Pantalar Arthrodesis (Combined Technique)			165	70	None	Excellent	0	0
**Er.12**	F	33			Distal	Inconclusive	Bone Transport			180	18	Pin site Infection	Excellent	1	0
**Er.13**	M	66			Distal	Inconclusive	Compression+ Arthrodesis			120	60	None	Excellent	1	1
**Ln.1**	F	38	21	3	Distal	Inconclusive	Compression+ Arthrodesis			165	34		Poor	5	4

**Ln.2**	M	55	14	10	Distal	Inconclusive	Compression			188	60		Good	6	0
**Ln.3**	M	41	5	4	Distal	Inconclusive	Resection+ Compression			138	45		Excellent	2,5	2
**Ln.4**	M	40	25	4	Distal	Inconclusive	Resection+ Compression			224	60		Excellent	3	1
**Ln.5**	M	62	60	13	Distal	Inconclusive	Compression+ Arthrodesis			307	27		Excellent	4	1
**Ln.6**	F	23	48	9	Distal	Inconclusive	Resection+ Compression			218	72		Excellent	3	0
**Me.1**	F	33	5	1		Inconclusive	Ilizarov 4 circles of distraction and compression without debridement	None	No	21w	38	Pin site infection	Excellent	0	0
**Me.2**	F	27	6	1		Inconclusive	Ilizarov 4 circles of distraction and compression without debridement	None	No	26w	26	Pin site infection	Excellent	0	0
**Me.3**	M	25	5	1		Inconclusive	Ilizarov 4 circles of distraction and compression without debridement	None	No	25w	25	Pin site infection	Excellent	0	0
**Me.4**	M	20	6	1		Inconclusive	Ilizarov 4 circles of distraction and compression without debridement	None	No	22w	24	None	Poor	0	2,5
**Me.5**	M	22	6	1		Inconclusive	Ilizarov 4 circles of distraction and compression	None	No	22w	25	Pin site infection	Excellent	0	0,5

							without debridement								
**Me.6**	M	24	5	1		Inconclusive	Ilizarov 4 circles of distraction and compression without debridement	None	No	24w	27	None	Good	0	0,8
**Si.1**	F	67	Inconclusive	Inconclusive	Distal	S. epidermidis, S. lugdensis, S. capitis	Masquelet, Locking plate	Cancellous iliac crest, allograft	No	210	Inconclusive	None	Inconclusive	Inconclusive	Inconclusive
**Si.2**	F	65	Inconclusive	Inconclusive	Proximal	S. epidermidis	Masquelet, Locking plate	Cancellous iliac crest, allograft	No	120	Inconclusive	None	Inconclusive	Inconclusive	Inconclusive
**Si.3**	F	88	Inconclusive	Inconclusive	Distal	S. warneri, S. epidermidis, MSSA, S. hominis	Masquelet, Locking plate	Cancelous iliac crest	No	510	Inconclusive	None	Inconclusive	Inconclusive	Inconclusive
**Si.4**	M	24	Inconclusive	Inconclusive	Distal	MRSA	Masquelet, Locking plate	Cancellous iliac crest, allograft	No	150	Inconclusive	None	Inconclusive	Inconclusive	Inconclusive
**Si.5**	F	78	Inconclusive	Inconclusive	Distal	MSSA	Masquelet, Locking plate	Cortical iliac crest, Allograft	No	180	Inconclusive	None	Inconclusive	Inconclusive	Inconclusive
**Si.6**	M	52	Inconclusive	Inconclusive	Distal	Strep agalactiae, S epidermidis, S simulans, E. cloacae	Masquelet, Plaster cast	Cancelous Iliac crest, femoral head	No	960	Inconclusive	None	Inconclusive	Inconclusive	Inconclusive
**Si.7**	M	64	Inconclusive	Inconclusive	Distal	MSSA	Masquelet, Locking plate	Cancellous iliac crest, allograft	No	Revision and union after 300	Inconclusive	Infection persist	Inconclusive	Inconclusive	Inconclusive
**Si.8**	M	49	Inconclusive	Inconclusive	Distal	MSSA	Masquelet, Locking plate	Cancelous iliac crest	No	Revision and union after 480	Inconclusive	Infection Persist	Inconclusive	Inconclusive	Inconclusive
**Si.9**	M	50	Inconclusive	Inconclusive	Distal	S epidermidis, S. auricularis	Masquelet, Plaster cast	Cancelous iliac crest	No	amputation	Inconclusive	Amputation	Inconclusive	Inconclusive	Inconclusive
**Si.10**	M	49	Inconclusive	Inconclusive	Distal	MSSA	Masquelet, External fixator	Femoral head	No	Ankle fusion	Inconclusive	Ankle fusion	Inconclusive	Inconclusive	Inconclusive

**Yo.1**	M	43	Inconclusive	5	Distal	Enterobacter cloacae	Removal of implants, debridement, 2nd stage Masquelet Tec, Definite plate	None	Inconclusive	52w	27	Recurrence of infection debridement and Ilizarov	Fair	4,8	0
**Yo.2**	M	61	Inconclusive	2	Distal	Acinetobacter baumanii	Removal of implants, debridement, 2nd stage Masquelet Tec, Intramedullary nail & augmented plate	None	Inconclusive	24w	24	Soft tissue defect, muscle flap, Intramedullary nail	Good	5,2	0
**Yo.3**	M	61	Inconclusive	1	Distal	MRSA	Removal of implants, debridement, 2nd stage Masquelet Tec, Intramedullary nail & augmented plate	None	Inconclusive	16w	12	None	Good	5,9	0
**Yo.4**	F	40	Inconclusive	1	Distal	Acinetobacter baumanii	Removal of implants, debridement, 2nd stage Masquelet Tec, Intramedullary nail & augmented plate	None	Inconclusive	16w	28	None	Good	4,5	0
**Yo.5**	M	50	Inconclusive	3	Distal	MRSA	Removal of implants,	None	Inconclusive	35w	15	None	Good	3,2	0

	M						debridement, 2nd stage Masquelet Tec, Intramedullary nail & augmented plate								
**Yo.6**	M	68	Inconclusive	1	Proximal	Acinetobacter baumanii, Enterobacter cloacae, Stenotrophomonas maltophilia	Removal of implants, debridement, 2nd stage Masquelet Tec, Definite plate	None	Inconclusive	20w	30	None	Excellent	5,6	0
**Yo.7**	M	64	Inconclusive	2	Distal	MSSA	Removal of implants, debridement, 2nd stage Masquelet Tec, Definite intramedullary nail	Massive	Inconclusive	24w	37	None	Good	4,1	0
**Yo.8**	M	33	Inconclusive	2	Distal	MRSA	Removal of implants, debridement, 2nd stage Masquelet Tec, Intramedullary nail & augmented plate	None	Inconclusive	20w	30	Soft tissue defect, muscle flap, Intramedullary nail & augmented plate	Excellent	10,2	0
**Yo.9**	M	36	Inconclusive	5	Distal	Pseudomonas	Removal of implants, debridement, 2nd stage Masquelet Tec,	None	Inconclusive	12w	12	None	Excellent	7,8	0

							Intramedullary nail & augmented plate								
**Yo.10**	M	59	Inconclusive	3	Proximal	MRSA	Removal of implants, debridement, 2nd stage Masquelet Tec, Definite plate	None	Inconclusive	20w	24	None	Excellent	3,5	0
